# No Chemopreventive Effect of Nonsteroidal Anti-Inflammatory Drugs on Nonmelanoma Skin Cancer: Evidence from Meta-Analysis

**DOI:** 10.1371/journal.pone.0096887

**Published:** 2014-05-14

**Authors:** Bo Zhang, Xiaolu Liang, Liaosha Ye, Yungui Wang

**Affiliations:** 1 Training Division, Third Military Medical Universality, Chongqing, China; 2 Department of medical administration, General Hospital of Beijing Military Command, Beijing, China; 3 Department of Ultrasonography, General Hospital of Beijing Military Command, Beijing, China; Duke Cancer Institute, United States of America

## Abstract

**Background:**

Nonmelanoma skin cancer (NMSC),which includes squamous cell carcinoma (SCC) and basal cell carcinoma (BCC), is the most common form of cancer, and its incidence is increasing. Nonsteroidal anti-inflammatory drugs (NSAIDs) have been reported to be chemopreventive for NMSC. However, the results from published studies were controversial.

**Methods:**

We searched the PubMed and Embase databases for relevant studies. Moreover, relevant reviews regarding the use of NSAIDs for NMSC patients were examined for potential inclusive studies. To measure the effects of NSAIDs, the relative risk (RR) was analyzed.

**Results:**

A Total of 8 studies were included in our meta-analysis. We found that NSAIDs use was not associated with a reduced risk of SCC or BCC under the random effects model (pooled RR  =  0.86, 95% CI, 0.73–1.02, P  =  0.085; pooled RR  =  0.94, 95% CI 0.85–1.04, P  =  0.266; respectively).

**Conclusion:**

From the included studies, we found no statistically significant chemopreventive effect on NMSC of NSAIDs. This finding warrants more prospective studies evaluating the relationship between NSAIDs and NMSC.

## Introduction

Nonmelanoma skin cancer (NMSC) is the most common form of cancer, and its incidence is increasing worldwide [1]–[3]. In the US, it is reported that over 1 million new skin cancers are diagnosed yearly, accounting for approximately 40% of all new cancer diagnoses [4]. The majority of skin cancer cases, with the most common types of NMSC being squamous cell carcinoma (SCC) and basal cell carcinoma (BCC) [5]. Numerous factors have been reported to contribute to the development of NMSC. Most importantly, ultraviolet radiation (UVR) exposure has been recognized as a direct cause of NMSC [6], and hence, sun avoidance and the use of broad spectrum sunscreens are recommended in order to block the initial steps of both photoaging and the carcinogenesis of NMSC. In addition to ultraviolet radiation exposure, other factors known to increase the risk of NMSC include exposure to thiopurines in patients with inflammatory bowel disease [7].

Chemopreventive effects of non-steroidal anti-inflammatory drugs (NSAIDs), which are usually indicated for the treatment of pain and inflammation, for NMSC, as well as for other types of cancers such as esophageal and gastric cancers, have been recently reported by several studies [8]–[12]. Similarly to their anti-inflammatory effects, NSAIDs act as chemopreventive agents by inhibiting the cyclooxygenase (COX) pathway.

The chemopreventive effects of NSAIDs, particularly aspirin, on NMSC have been previously demonstrated using animal experiments [13], [14], and recently, a number of human studies have been conducted to assess the chemopreventive effects of NSAIDs on NMSC, with varingresults. Although some of these studies suggested that NSAID use may decrease the risk of SCC or BCC [15], others found no effect, resulting in uncertainty and controversy regarding the relationship between NSAID use and the risk of NMSC [16]. Moreover, no meta-analysis that analyzing and summarizing the evidence on the chemopreventive effects of NSAIDs for NMSC has been performed to date. Hence, we here performed a meta-analysis of relevant epidemiologic studies and randomized controlled trials to evaluate whether the chemopreventive effect of NSAIDs on NMSC exists quantitatively.

## Methods

### Search strategy

Related studies in English on human participants were identified by searching multiple electronic databases, including PubMed (from 1976 to September 2012) and Embase (from 1966 to September 2012). The search algorithm was generated as (“nonsteroidal anti-inflammatory drugs” or “aspirin” or “celecoxib” or “ibuprofen” or “diclofenac” or “piroxicam” or NSAIDs) and (“nonmelanoma skin cancer” or “squamous cell carcinoma” or “basal cell carcinoma” or NMSC). Furthermore, we also manually searched the reference lists of relevant review articles. English-language literatures were eligible for inclusion in our analysis, regardless of publication status (published, unpublished, in press, or in progress).

### Criteria for inclusion

Studies were included if they met all of the following inclusion criteria: (i) the association of exposure to NSAIDs with the incidence of NMSC was studied; and (ii) the relative risk (RR), odds ratio (OR), or hazard ratio (HR) with the corresponding 95% confidence intervals (95% CIs) was provided.

### Data extraction

Using a standardized data collection form, the literature search and data extraction were undertaken independently by 2 investigators (Zhang B and Ye LS). Disagreements were resolved by a third reviewer (Wang YG) after referring to the original articles.

The following information was collected from the identified studies: publication information (title, first author's name, and publication year), study characteristics (study design, study region, endpoint index, and regimen), and outcome for the whole study population.

### Statistical analysis

We assessed the effect of NSAIDs on NMSC from the included studies. RR was used as a measure for the relationship between NSAIDs and the risk of NMSC. And differences observed between groups were expressed as RR with the corresponding 95% CI. Owing to the fact that the incidence of NMSC was low, we deemed that the OR and HR approximates the RR. Individual RRs and 95% CIs were extracted or calculated initially, and then log RR and its corresponding standard error were estimated for pooling. The χ^2^ test and I^2^ statistic were employed to assess variability across studies attributable to heterogeneity beyond chance [17]. A P-value>0.10 for the χ^2^ test and an I^2^ value <25% were interpreted as signifying low-level heterogeneity. When there was no statistically significant heterogeneity, a pooled effect was calculated using a fixed-effect model; otherwise, a random effect model was employed. A random effects model assumes that individual studies estimate different treatment effects, and in order to make some sense of these different effects, it is assumed that they have a distribution with a central value and some degree of variability. That is, under random effects modeling, the true effects in the studies are assumed to have been sampled from a distribution of true effects.

For one trial comparing different groups, we first combined the group together under the fixed model to get a pooled estimate of a single study [18]. For one study comparing different NSAID use groups with non-NSAID users, we combined the groups to create a single pair-wise comparison by incorporating the effect size and its variance of the current NSAID use group versus the control with those of the past NSAID use group by pair-wise comparison [19]. We moreover undertook subgroup analyses according to the different varieties of NSAIDs. Sensitivity analysis was performed to test the stability of the results.

All reported P values were two-sided and P values <0.05 were regarded as statistically significant. Statistical analysis was conducted with STATA 11.0 (Stata Corporation, Lakeway, TX, USA) and the freeware Review Manager (version 5.1 for Windows, Cochrane Collaboration, Oxford, UK, 2011).

## Results

### Study characteristics

**Figure 1:**
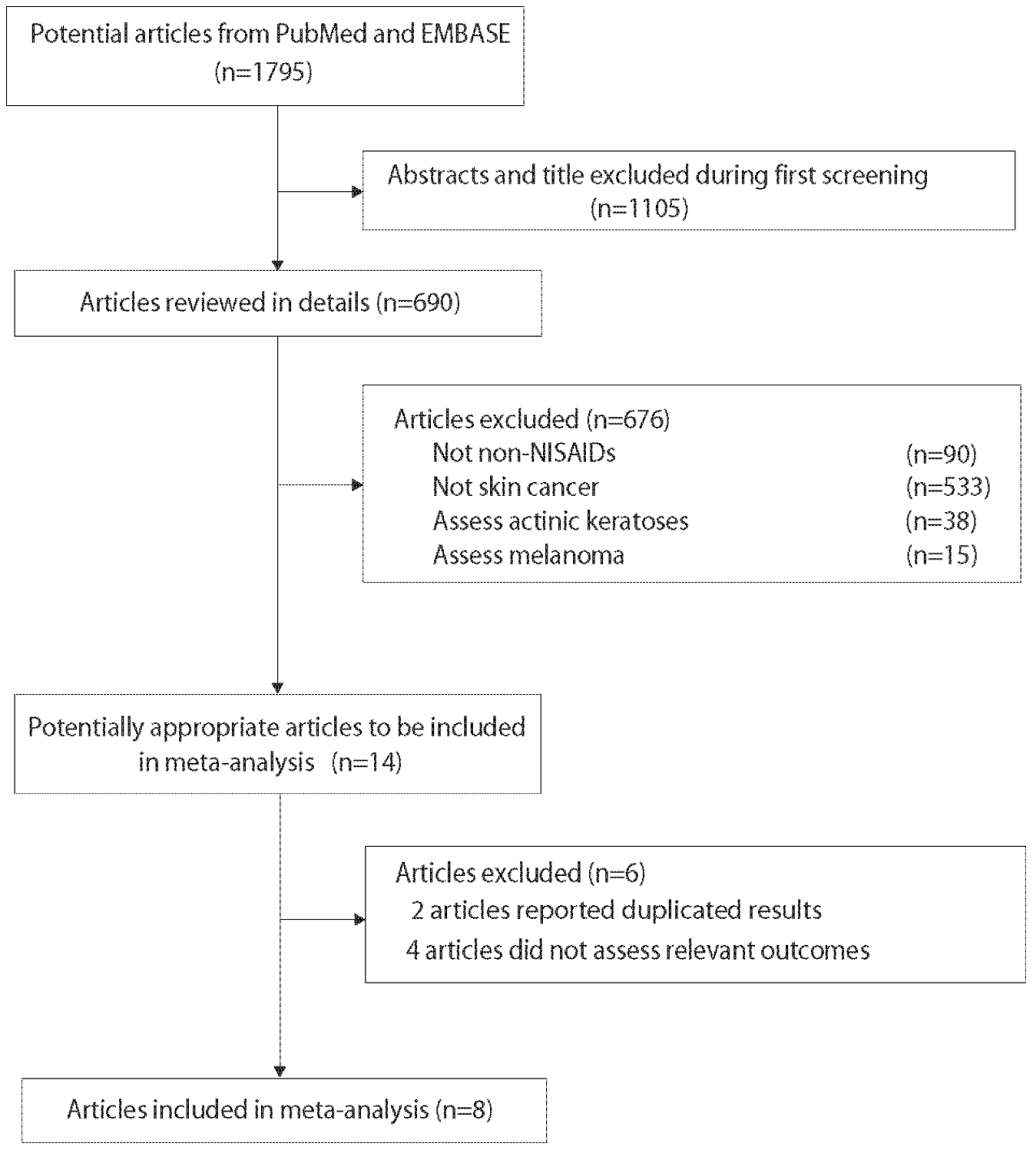
Flow chart of the literature search.

A total of 1795 potentially relevant studies were identified by the search strategy defined above, out of which 1781 were excluded (Fig. 1). During the initial screening, we read the titles and abstracts of all potential studies. A total of 1105 studies which were reviews, short communications, or conducted in animals were excluded. Fourteen studies were reviewed as potentially relevant studies, out of which 6 were excluded on the basis of the established exclusion criteria. Finally, 8 studies, including 1 randomized controlled trial and 7 observational studies were included in the analysis (Table 1) [15], [16], [19]–[24].

**Table 1:** Characteristics of the included studies.

No	First author	Region	Year	Study design	Sample size	Index	Regimen	outcome
1	Maria V. Grau	US	2006	Case-control study	702 cases; 1019 controls	Adjusted OR	NSAIDs	SCC, BCC
2	Mary C. Clouser	US	2009	Cohort study	321 any NSAID uesers; 1081 no NSAID users	Adjusted HR	NSAIDs, aspirin only, non-aspirin	SCC, BCC
3	Maryam M. Asgari	US	2010	Case-control study	415 with SCC; 415 controls	Adjusted OR	NSAIDs, aspirin only, non-aspirin, celecoxib	SCC
4	Craig A. Elmets	US	2010	RCT	122 celecoxib group;118 placebo group	RR	celecoxib	SCC, BCC
5	Dorothea C. Torti	US	2011	Case-control study	535with SCC; 487 with BCC; 462 controls	Adjusted OR	NSAIDs, aspirin only	SCC, BCC
6	Elizabeth K. Cahoon	US	2011	Cohort study	2291 with BCC; 55922 without BCC	Adjusted HR	NSAIDs	BCC
7	Johannesdottir SA	Denmark	2012	Case-control study	1974 with SCC; 13316 with BCC; 178655 controls	Adjusted OR (incidence rate ratio)	NSAIDs, celecoxib	SCC, BCC
8	J. M. Jeter	US	2012	Cohort study	92125 Caucasian women	Adjusted RR	aspirin only, non-aspirin	SCC, BCC

In J. M. Jeter's study [19], they compared the effect of NSAID on BCC or SCC for past users and current users with non-users, separately. We firstly combined them together under fixed model to get a pooled estimate.

### Any NSAIDs versus non-NSAID users

**Figure 2:**
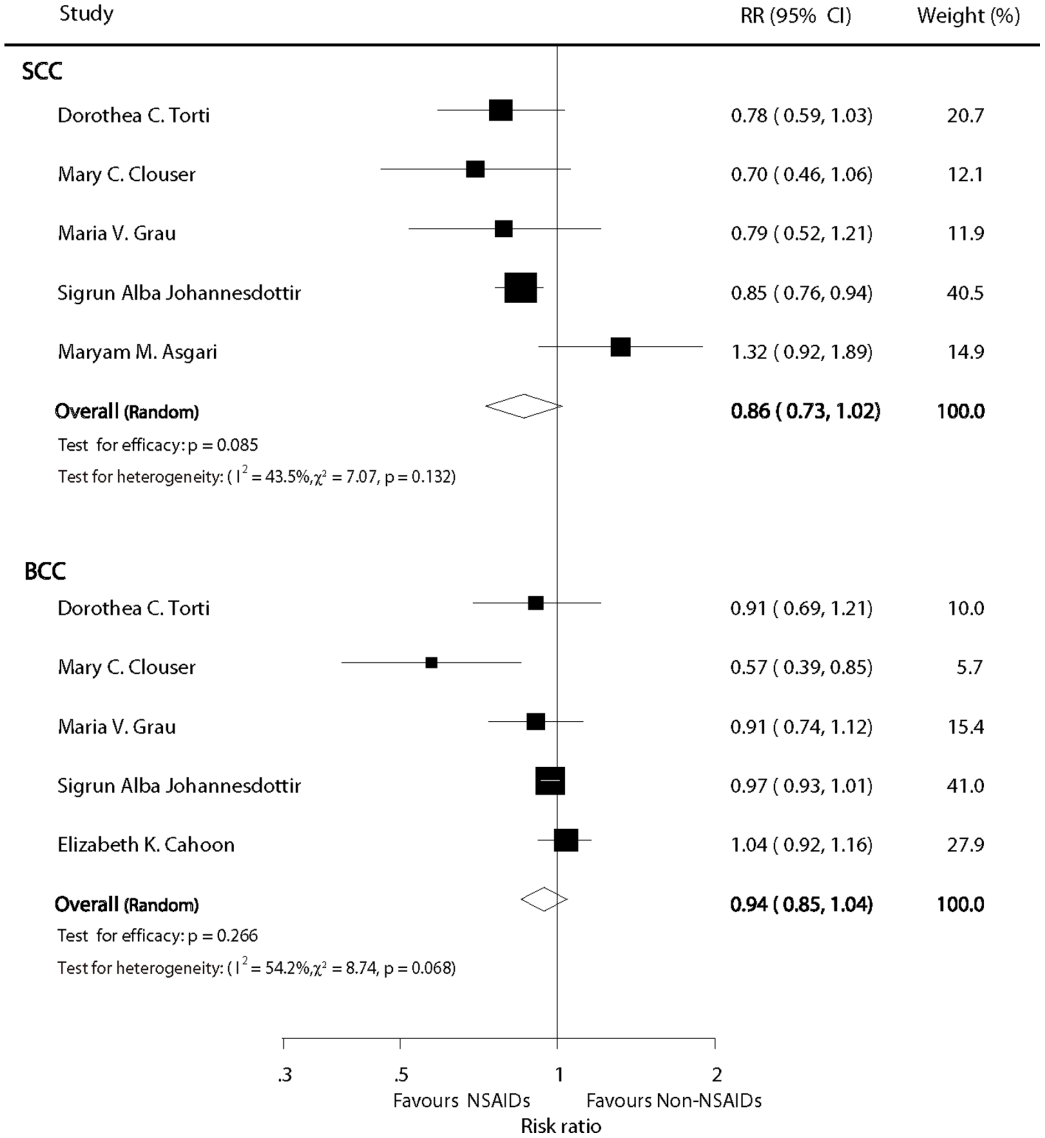
Forest plot of any NSAID versus non-NSAID users.

Information about the chemopreventive effects of NSAIDs was available in 5 studies. The estimated pooled RR did not show a statistical significantly chemopreventive effect on SCC for patients who received NSAIDs compared with non-NSAIDs users (pooled RR  =  0.86; 95% CI: 0.73–1.02; P  =  0.085; Fig. 2), and the result was similar for BCC (RR  =  0.94; 95%CI: 0.85–1.04; P  =  0.266; Fig. 2). It is possible that there may have been substantial heterogeneity in the RRs for both BCC and SCC from the individual trials (I^2^  =  43.5%, P  =  0.132 and I^2^  =  54.2%, P  =  0.068, respectively Fig.2), and we thus incorporated the data into the random effects model.

### Aspirin versus non-NSAID users

**Figure 3:**
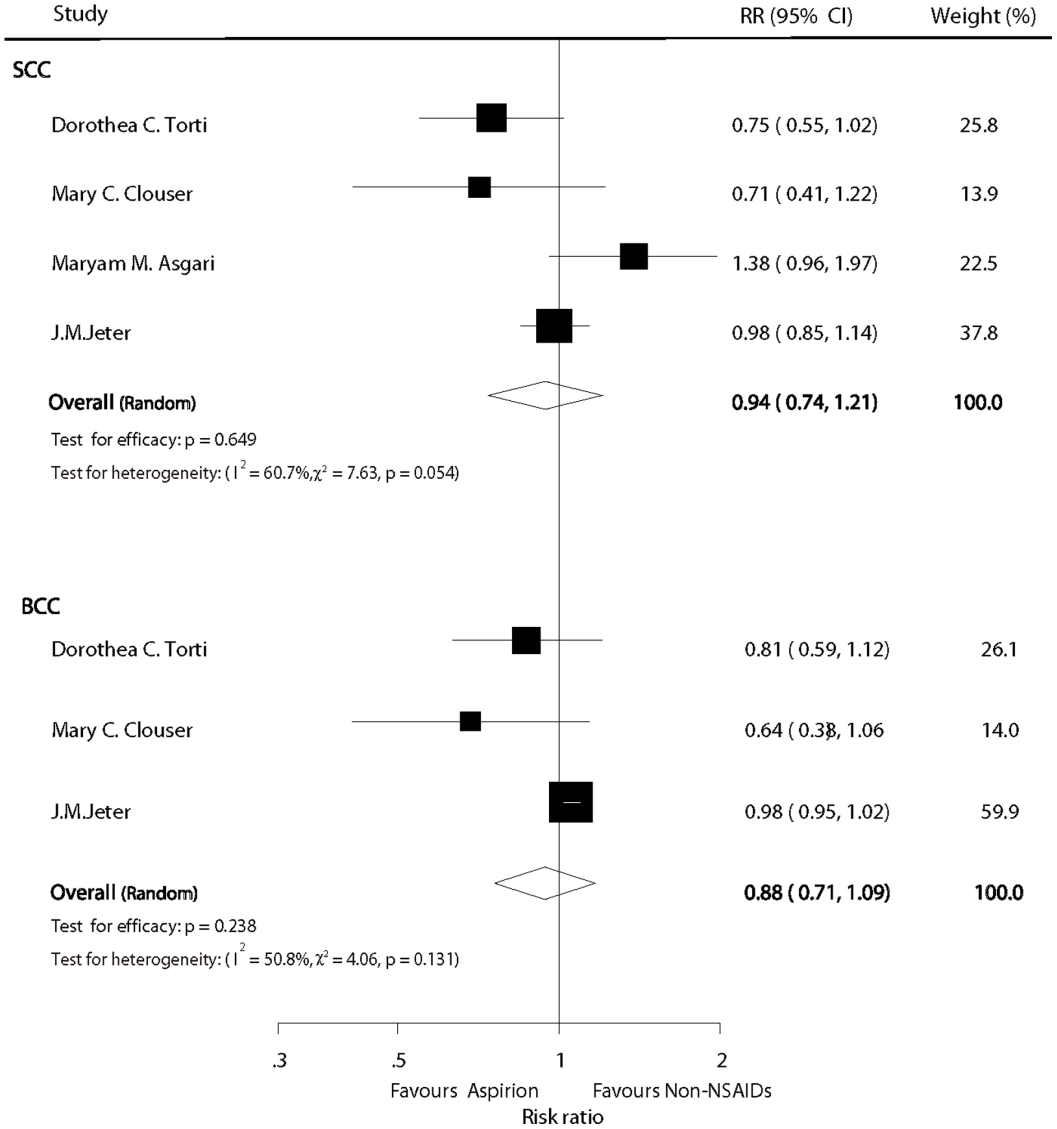
Forest plot of aspirin versus non-NSAID users.

Four and 3 studies provided RRs for aspirin vs. non-NSAID use on SCC and BCC, respectively. Overall, the pooled analyses demonstrated that there were no statistically significant chemopreventive effects of aspirin on either SCC or BCC compared with non-users (RR  =  0.94, 95% CI: 0.74–1.21, P  =  0.649; and RR  =  0.88, 95%CI: 0.71–1.09, P  =  0.238, respectively; Fig. 3) However, as some heterogeneity (I^2^  =  60.7%, P  =  0.054; and I^2^  =  50.8%, P  =  0.131, respectively; Fig. 3) was observed, the random effect model was used for the analyses.

### Non-aspirin NSAIDs versus non-NSAID users

**Figure 4:**
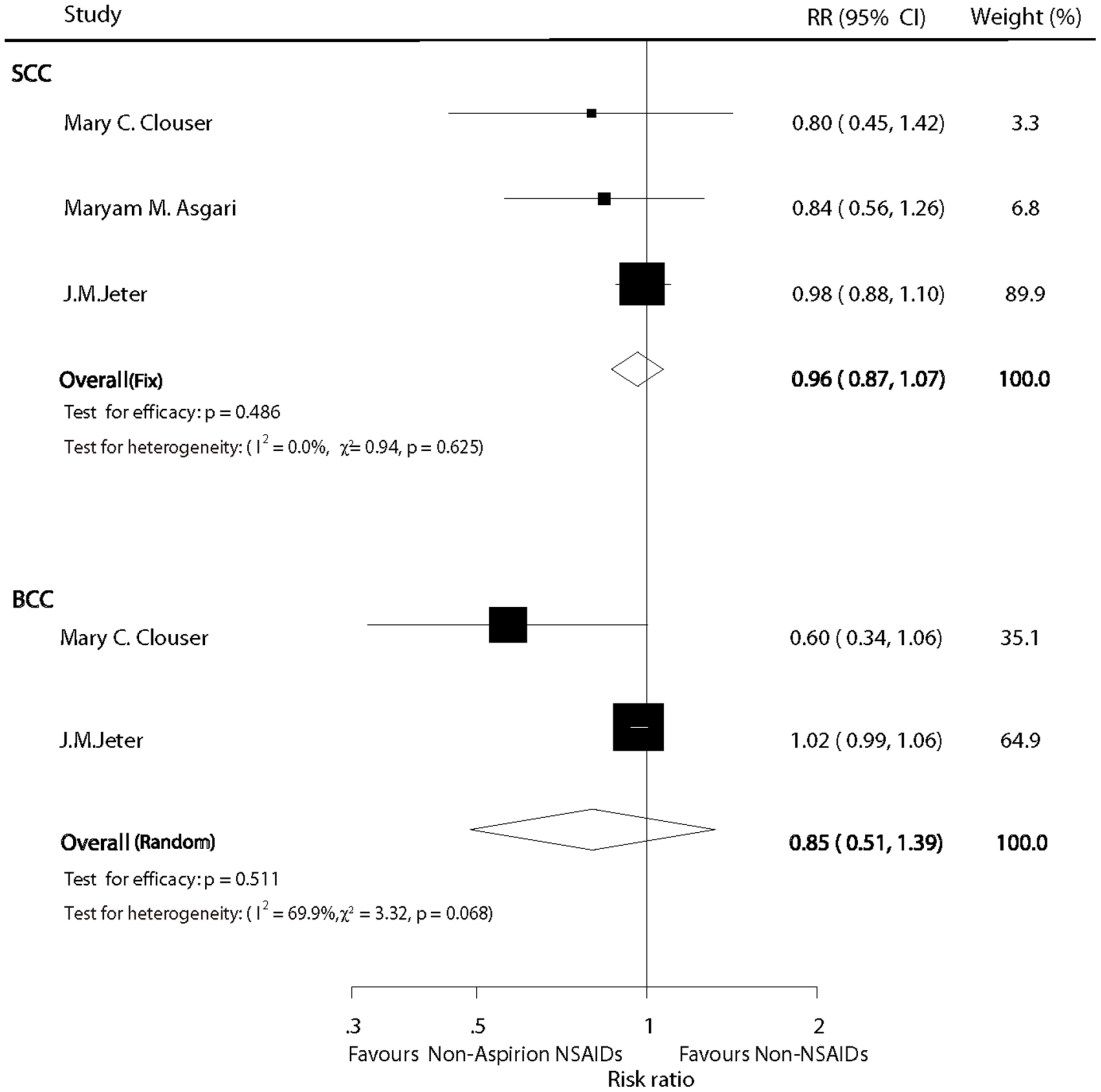
Forest plot of non-aspirin NSAID versus non-NSAID users.

On the basis of included studies, our meta-analysis did not identify a statistical significant chemopreventive effect on SCC for patients who received non-aspirin NSAIDs compared with non-users (pooled RR  =  0.96; 95% CI: 0.87–1.07; P  =  0.486) under a fixed model (I^2^  =  0%; P  =  0.625; Fig. 4). For BCC, the combined estimate of the RR was 0.85 (95%CI: 0.51–1.39; P  =  0.511) under a random model (I^2^  =  69.9%; P  =  0.068; Fig. 4)

### Celecoxib versus non-NSAIDs users

**Figure 5:**
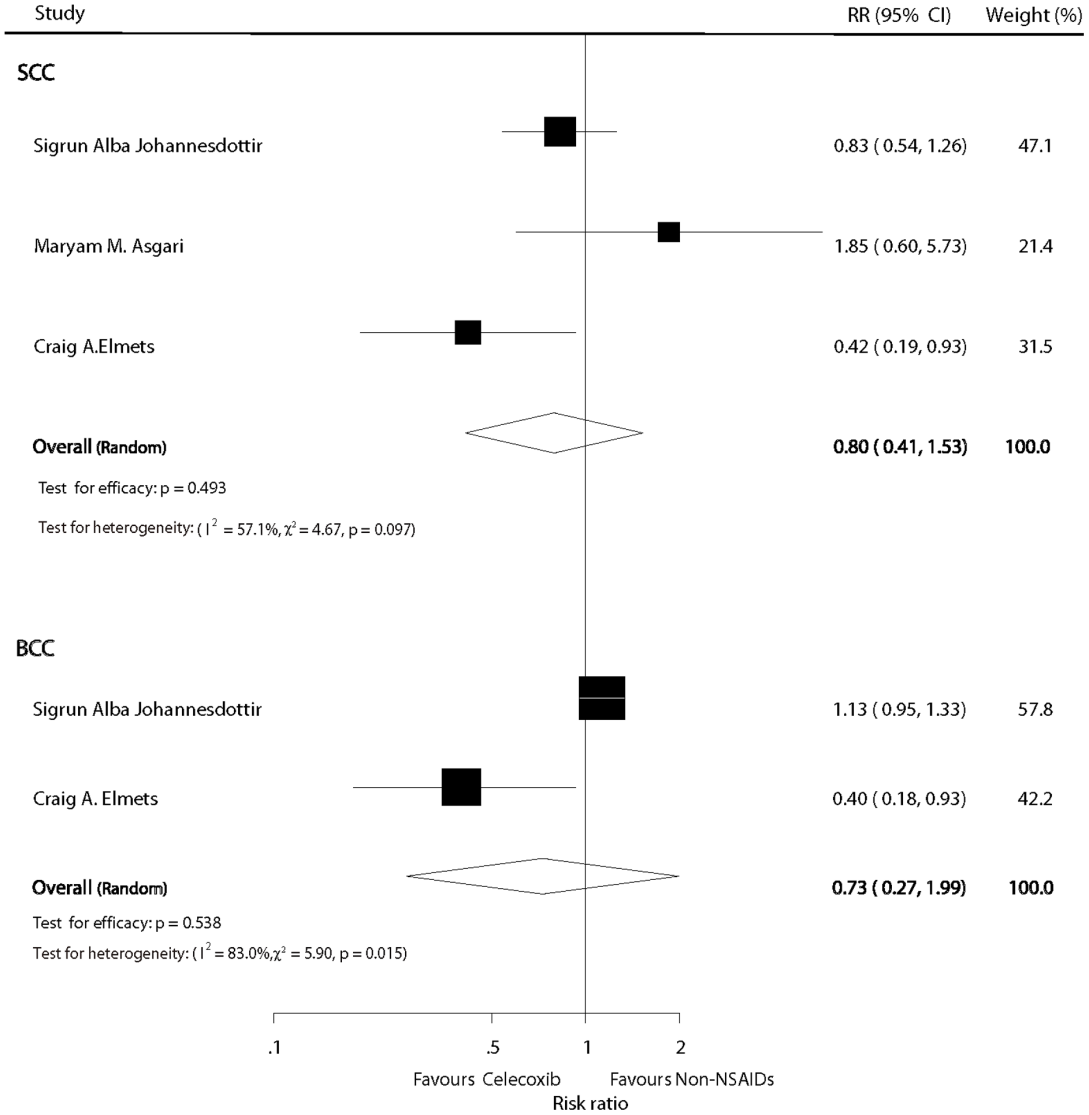
Forest plot of celecoxib versus non-NSAID users.

The pooled results did not show a statistical significant chemopreventive effect on SCC for patients who received celecoxib compared with non-users (pooled RR  =  0.80; 95% CI: 0.41–1.53; P  =  0.493) under a random model (I^2^  =  57.1%; P  =  0.097; Fig. 5). Similarly, the combined estimate of the RR for BCC was 0.73 (95%CI: 0.27–1.99; P  =  0.538) under a random model (I^2^  =  83%; P  =  0.015; Fig. 5).

### Sensitivity analysis

Of the studies considered, we found the result from Maryam M. Asgari's study was quite different from other studies. Therefore we performed a sensitivity analysis to exclude this study to testify the stability of the results. It did not reduce the p-value to below 0.05 except in regards to the chemopreventive effect of NSAIDs on SCC (pooled RR  =  0.83; 95% CI: 0.76–0.91; Table 2), and the point estimates were farther away from 1 (null).

**Table 2:** Sensitivity analysis after excluding the study by Asgari et al.

Index	RR(95%CI)	Heterogeneity(I^2^)	P value for Heterogeneity test
NSAIDs(SCC)	0.83(0.76,0.91)	0.0%	0.775
Asprion(SCC)	0.87(0.70,1.07)	38.7%	0.196
Celecoxib(SCC)	0.64(0.34,1.23)	54.6%	0.138

## Discussion

To the best of our knowledge, this is the most recent report to investigate the chemopreventive effects of NSAIDs on NMSC, and the first meta-analysis on the topic. We carried a broad search of manually reviewed databases, which yielded eight studies that met out inclusion criteria. Statistical analysis of these studies showed no chemopreventive effect of NSAIDs on SCC or BCC. In addition, based on the included studies, we also explored the chemopreventive effect of aspirin, non-aspirin NSAIDs, and celecoxib, but found no chemopreventive effects of any of these regimens on SCC or BCC. The chemopreventive actions of NSAID in NMSC do not echo similar findings in other types of cancers, such as, etc, and besed on the present analysis, we conclude that there are no significant chemopreventive effects of NSAIDs on NMSC.

The included studies had different characteristics. Johannesdottir et al. used data of all reported cases in northern Denmark during 1991–2009. They included 1974 SCCs and 13316 BCCs and approximately 10 population controls for each case, and reported that NSAID use was associated with a decreased risk of SCC but not BCC [15]. Torti et al conducted a case-control study, which included 535 subjects with SCC, 487 with BCC, and 462 control subjects, and found that NSAIDs use, and particularly aspirin, was associated with a reduced risk of SCC, but not BCC. In addition to exploring the relationship between NSAIDs and NMSC, the authors moreover reported that certain genes could affect the relationship, with decreased ORs observed in a subset of SCC tumors positive for p53 and with loss of heterozygosity of *PTCH*. However, they furthermore stated that self-reported drug use was a limitation of their study [24]. Asgari et al. conducted their case-control study using the Kaiser Permanente Northern California population. They investigated whether NSAID use was associated with a reduced risk of SCC, but found no association between the two. However, the small sample size used was a major limitation of this study, with only 86 cases and 187 controls being included [16]. Clouser et al. retrieved data from the SKICAP-AK trial, which was a randomized controlled trial with the purpose of testing the association between daily supplementation of retinol and the incidence of skin cancer. In this study, the protective effects of NSAIDs on the development of a first case of BCC or SCC were observed. Meanwhile, the authors also demonstrated that short-term NSAID use was more protective than long-term use, which is contrarythat long-term use is more efficient. However, it should be noted that, similar to the study by Torti et al., self-reported drug use was also a limitation in this study [21]. Conversely, Jeter et al. performed a cohort study and demonstrated that aspirin and other NSAIDs were not associated with a lower risk of SCC, or BCC in women. The strengths of their study included the prospective design and large number of skin cancer cases, whereas the major limitation was that it included only Caucasian female nurses, and the results may thus not be representative of the general population [19]. Elmets et al. performed a randomized controlled trial to investigate the chemopreventive effect of celecoxib on NMSC, and observed the chemopreventive effects of NSAIDs on both BCC and SCC [22]. On the other hand, Cahoon et al. conducted a large cohort study investigating the association between NSAID use and BCC, and did not observe a relationship between the two [20]. Lastly, Grau et al. used data from the Skin Cancer Prevention Study, a randomized controlled trial, to investigate the effects of NSAIDs on the recurrence of NMSCs. Their study suggested a weak and inconsistent chemopreventive effect of NSAIDs on NMSC, and the authors reported that the major limitation of the study was the lack of data regarding the doses and duration of NSAID use in the study subjects [23].

The strength of our present study lies in the detailed analysis of the chemopreventive effects of NSAIDs on NMSC. We not only analyzed the chemopreventive effect of NSAIDs as a whole, but also performed separate sub-group analyses for aspirin, non-aspirin NSAIDs, and celecoxib. In addition, we analyzed SCC and BCC separately as well, rather than analyzing all NMSCs grouped together.

The main purpose of the present meta-analysis was to present and analyze all available evidence in a systematic, quantitative, and unbiased fashion. However, several technical limitations of this meta-analysis should be acknowledged. First, the null result was reached for both SCC and BCC for participants taking NSAIDs. This might be due to the small number of included studies and the inter-study heterogeneity. Further original studies must be included and combined to increase the power of meta-analysis. Second, this meta-analysis used data mainly from observational studies which were unable to control for the same important potential confounders andthe different demographics of the study populations may lead to valid differences in the magnitude and direction of the investigated association. Third, different indices, such as adjusted RR and adjusted HR, were used to describe the chemopreventive effects of NSAIDs on NMSC. In this study, although we used RR to combine all these indices, the pooled RR cannot be regarded as a fully accurate index to measure the effect. Fourth, most of the included studies were conducted in the US, with similar studies rarely reported in other regions. Lastly, heterogeneity among studies may represent another limitation of our meta-analysis, and to avoid this, we applied a random effect model that takes possible heterogeneity into consideration. Owing to the limitations of this meta-analysis, further studies are required to confirm the clinical importance of our findings.

In summary, this comprehensive meta-analysis demonstrates that there is no statistically significant chemopreventive effect of NSAIDs on NMSC. Owing to the intrinsic limitations of observational studies, further studies are needed to investigate whether this chemopreventive effect exists or not.

## Supporting Information

Checklist S1 **PRISMA Checklist.**
(DOC)
